# Enhancement on migration and biodegradation of *Diaphorobacter* sp. LW2 mediated by *Pythium ultimum* in soil with different particle sizes

**DOI:** 10.3389/fmicb.2024.1391553

**Published:** 2024-05-22

**Authors:** Jialu Li, Mei Hong, Jing Lv, Rui Tang, Ruofan Wang, Yadong Yang, Na Liu

**Affiliations:** ^1^Key Laboratory of Groundwater Resources and Environment, Ministry of Education, College of New Energy and Environment, Jilin University, Changchun, China; ^2^National and Local Joint Engineering Laboratory for Petrochemical Contaminated Site Control and Remediation Technology, Jilin University, Changchun, China; ^3^School of Environmental Science and Engineering, Jiangsu Engineering Research Center of Biomass Waste Pyrolytic Carbonization & Application, Yancheng Institute of Technology, Yancheng, China; ^4^Department of Ecology, College of Life Science and Technology, Jinan University, Guangzhou, Guangdong, China

**Keywords:** bacterial migration, growth direction, heterogeneous soil, hyphae, biodegradation of phenanthrene

## Abstract

**Introduction:**

The composition and structure of natural soil are very complex, leading to the difficult contact between hydrophobic organic compounds and degrading-bacteria in contaminated soil, making pollutants hard to be removed from the soil. Several researches have reported the bacterial migration in unsaturated soil mediated by fungal hyphae, but bacterial movement in soil of different particle sizes or in heterogeneous soil was unclear. The remediation of contaminated soil enhanced by hyphae still needs further research.

**Methods:**

In this case, the migration and biodegradation of *Diaphorobacter* sp. LW2 in soil was investigated in presence of *Pythium ultimum*.

**Results:**

Hyphae could promote the growth and migration of LW2 in culture medium. It was also confirmed that LW2 was able to migrate in the growth direction and against the growth direction along hyphae. Mediated by hyphae, motile strain LW2 translocated over 3 cm in soil with different particle size (CS1, 1.0–2.0 mm; CS2, 0.5–1.0mm; MS, 0.25–0.5 mm and FS, <0.25 mm), and it need shorter time in bigger particle soils. In inhomogeneous soil, hyphae participated in the distribution of introduced bacteria, and the total number of bacteria increased. *Pythium ultimum* enhanced the migration and survival of LW2 in soil, improving the bioremediation of polluted soil.

**Discussion:**

The results of this study indicate that the mobilization of degrading bacteria mediated by *Pythium ultimum* in soil has great potential for application in bioremediation of contaminated soil.

## 1 Introduction

Soil is a major environmental sink for hydrophobic organic contaminants (HOCs) (Nam et al., 1998; Collins et al., 2013). HOCs tend to sorb to soil particulates due to their low solubility (Zeng et al., 2010). Wild and Jones (1995) reported that over 90% PAH burden resided in soil in UK, and the complex soil structure causes non-homogeneous distribution of HOCs (Gu et al., 2017). Biodegradation is often considered as one of the effective methods for contaminated soil remediation. Solids in soil are always arranged in a very complex spatial manner and form tortuous pore spaces. Soil heterogeneity can arise and persist without being mixed well. The bacterial transport in unsaturated soils across macro-scale distance (>10 mm) was primarily driven by water flow and reduced with decreasing water contents and water flow velocities (Or et al., 2007). Griffin and Quail (1968) reported that no movement of *Pseudomonas aeruginosa* was detected at water contents below 28%. Moreover, motile strains only spread 6.4 mm in vertical direction in soil with water content of 13.3% (w/w) after 14 days (Turnbull et al., 2001). Bosma et al. (1996) calculated the average distances between bacterial microcolonies in soil at a range of 50–100 μm. Effective contact between microorganisms and pollutants is a prerequisite for biodegradation. The difficulty of microbial migration in complex soil environments limits the removal of pollutants.

The interactions between fungi and bacteria widely prevail in a variety of habitats, especially in soil (de Menezes et al., 2017; Deveau et al., 2018). Kohlmeier et al. (2005) reported that hydrophilic *Fusarium oxysporum* Fo47 bridged air gaps and provided water channels for the migration of *Achromobacter* sp. SK1. After that, several literature studies reported the bacterial movement mediated by hyphae. Warmink and Elsas (2009) found that the attached soil bacteria moved with growing hyphae and formed biofilms around hyphae. Effects of bacterial type three secretion system and hyphal surface receptors on bacterial migration along hyphae also have been investigated (Vila et al., 2016; Yang et al., 2016). Worrich et al. (2016) proposed that fungal mycelia could maintain the microbial growth and biodegradation even at low osmotic and matric potentials. In addition to microorganisms, soil properties were also important factors affecting bacterial migration. Nazir et al. (2010a) found that *Lyophyllum* sp. strain Karsten alleviated pH pressure in acid soil and enhanced bacterial survival. Higher moisture content and pH in acidic soil are beneficial for hyphal-mediated bacterial migration (Yang et al., 2018).

Except for pH and moisture content, soil particle size was also an important soil characteristic, but the bacterial migration mediated by mycelium in soils with different particle sizes was still unclear. Particle size of natural soil was uneven, and behavior of motile bacteria mediated by mycelium in heterogeneous soil was also unknown. Research on the enhanced biodegradation of phenanthrene in contaminated soil by hyphae is also very limited. In previous study, we have demonstrated that naphthalene- and phenanthrene-degrading *Diaphorobacter* sp. LW2 was able to migrate across air gaps with the hyphae of *Pythium ultimum* (Li et al., 2022). We chose these two microorganisms to investigate the bacterial migration in homogeneous and heterogeneous soil with different particle sizes mediated by mycelium, as well as the biodegradation of phenanthrene-contaminated soil with different particle sizes. First, the effect of *Pythium ultimum* on the biodegradation and growth of LW2 was studied. Next, the migration behavior of LW2 with fresh or old hyphae was studied, and the diffusion and distribution of bacteria introduced at the hyphal tip and end (growth starting point) were investigated. Then, bacterial migration mediated by hyphae in soil with different particle sizes was examined, and the distribution of introduced bacteria in heterogeneous soil in the presence of mycelium was explored. Finally, the effect of additional *Pythium ultimum* on the biodegradation of phenanthrene in soil with different particle sizes by LW2 was studied. We hope that the experimental results provided the theoretical basis for the bioremediation of contaminated soil.

## 2 Materials and methods

### 2.1 Experimental materials

#### 2.1.1 Organisms

Fungi, oomycete *Pythium ultimum,* with hydrophilic mycelia (collection number 37386) was purchased from the China Agricultural Culture Collection of China (Beijing, China). *Pythium ultimum* was cultivated at 28°C on solid medium of potato dextrose agar (PDA).

Naphthalene- and phenanthrene-degrading bacteria, *Diaphorobacter* sp. LW2, were isolated from the aged PAH-contaminated soil and had been proven to be able to move via *Pythium ultimum* mycelia. LW2 was cultivated in Luria–Bertani (LB) medium, harvested, and washed three times with 0.01 M PBS. Bacterial suspension used in mobilization experiment contained approximately 1.21 × 10^8^ CFU ml^−1^. Bacteria were quantified as colony forming units (CFUs) on LB solid medium containing 200 mg L^−1^ actidione to prevent fungal growth.

#### 2.1.2 Soil

Experiment soil was collected from Shandong province, China, and was sieved into four types according to granular sizes: coarse sand-1 (CS1, 1.0–2.0 mm), coarse sand-2 (CS2, 0.5–1.0 mm), medium sand (MS, 0.25–0.5 mm), and fine sand (FS, <0.25 mm). The pH values of CS1, CS2, MS, and FS were found to be 8.05–8.21. The initial water content and organic carbon content of these samples are shown in Supplementary Table S1. All soil samples were sterilized for three times with 30 min each time to ensure sterility. Soil samples were mixed with 5% (w/w) wheat bran to support hyphal growth and were adjusted to be with water content of ~10 wt%.

### 2.2 Biodegradation and growth of LW2 affected by *Pythium ultimum* hyphae

*Pythium ultimum* growing on PDA plates was inoculated into 24-well plates. In total, 1.5 mL PDB medium was added in each hole. After 3 days, the mycelium slices with a diameter of approximately 1.6 cm and a dry weight of approximately 2 mg were obtained. The mycelium was washed with PBS buffer three times, and part of it was used as inactivated mycelium after being autoclaved at 121°C for 15 min.

Due to the low solubility of phenanthrene in water, a concentration of 1 mg L^−1^ of phenanthrene was set in this experiment to avoid the interference of undissolved phenanthrene with bacterial growth and mycelium adsorption tests. Overall, 0.5 mL bacterial suspension and two inactivated or non-inactivated mycelium pieces were added to a sterile brown glass bottle containing 9.5 mL phenanthrene-MSM medium and cultured for 72 h at 25°C. Treatments with only the addition of bacteria or mycelium were considered as the controls. Overall, 0.5 mL PBS buffer was added to the groups without LW2 to avoid volume differences. The whole residual content of phenanthrene and OD_600_ of LW2 were tested at 0, 2, 4, 8, 10, 12, 24, 48, and 72 h. The concentration in water and whole residual content of phenanthrene were examined in groups adding only mycelium.

The extraction of phenanthrene is consistent with the report by Gu et al. (2016). The concentration of phenanthrene was measured using high-performance liquid chromatography (HPLC) (Li et al., 2022).

### 2.3 Influence of fresh or old hyphae on bacterial migration along with hyphae

**Figure 1:**
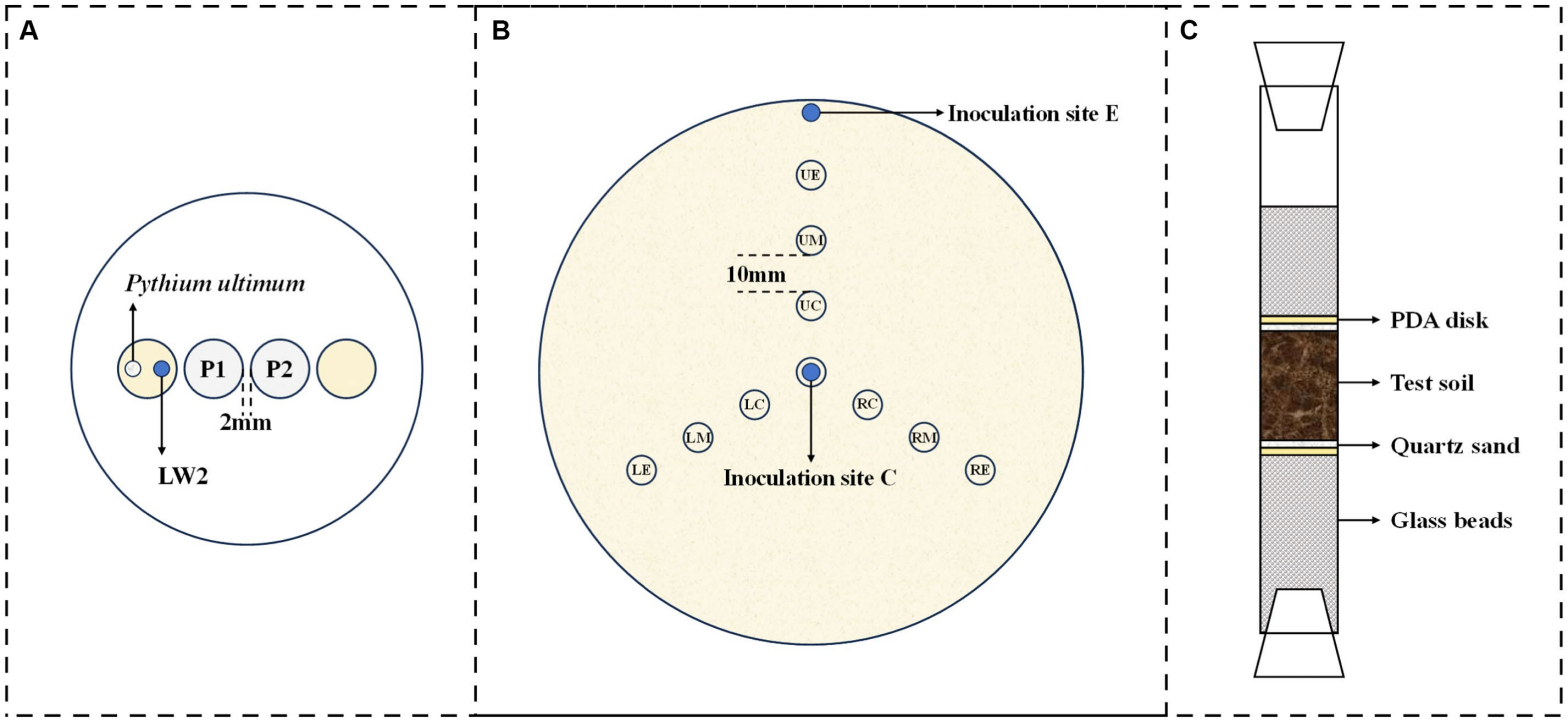
Schematic diagrams for microorganism migration experiments (**(A)**, migration of *Diaphorobacter* sp. LW2 affected by fresh or old *Pythium ultimum* hyphae; **(B)**, bacterial distribution in mycelial network; and **(C)**, bacterial migration in soil).

Fresh (newly grown) or old (grown over 7 days) hyphae were inoculated in Petri dishes containing PDA, and the diameter of mycelium coverage was regarded as an evaluation indicator for the hyphal growth. Bacterial migration along with fresh or old hyphae was tested. In brief, *Pythium ultimum* was introduced on the PDA disk of left side (Figure 1A) and had fully overgrown the system (approximately 4 days), and 10 μL of bacterial suspension was added. For the system that was pre-inoculated before 11 days, hyphae with excessive growth for 7 days were considered as old hyphae. After LW2 inoculation for 3 days, bacterial cells on agar disks at P1 and P2 positions were measured. Fungus-associated bacteria were treated by both vortexing for 60 s and ultrasonication of two times for 30 s, as previously reported (Wick et al., 2007). After appropriately diluted, the bacterial suspension was spread on LB agar containing 200 mg L^−1^ actidione following incubation, and colonies were enumerated. All tests were set up with triplicates.

### 2.4 Bacterial migration starting from the end or tip of the hyphae

The effect of bacterial inoculation location on the diffusion and distribution of LW2 in *Pythium ultimum* mycelium was investigated. Specifically, *Pythium ultimum* was introduced in the center of PDA plates (diameter: 150 mm), and 100 μL of bacterial suspension was added at center or edge of the plate when the hyphae reached the edge of the plate. The bacteria inoculated at the center of the plate were equivalent to being introduced at the end of the hyphae, while the bacteria inoculated at the edge of the plate corresponded to being introduced from the tip of the hyphae. At set times (2 h and 48 h after bacterial inoculation), small samples were recovered, by punching out, from the marked sites, as shown in Figure 1B. All samples were treated to quantify bacterial cells, as described in *2.3*. The adjacent sampling positions were spaced 10 mm apart, with an angle of 120 ° in the upper, left, and right directions. For each experimental treatment, three replicates were used. Controls received no bacteria or *Pythium ultimum*.

### 2.5 The bacterial mobilization by *Pythium ultimum* in soil with different particle size

The bacterial mobilization via *Pythium ultimum* mycelia was studied in laboratory systems mimicking air-filled soil environment, as shown in Figure 1C. The ends of the columns (length: 20 cm; diameter: 1.6 cm) were closed with breathable silicone test tube plugs, and 6.0 g glass beads (diameter: 0.4 cm) were placed at the bottom of the columns in purpose of supporting round PDA disks (diameter: 1.6 cm; thickness: 0.2 cm) covered by mycelium. Overall, 100 μL of bacterial suspension was inoculated onto the surface of PDA disks at the bottom simultaneously. The columns were filled with soil of different particle sizes, 1.5 cm or 3 cm thick, and 0.5 g of quartz sand above and below was to separate the PDA disks from the experimental soil. The glass columns were placed at constant temperature and humidity for inoculation, and the bacteria around upper glass beads were quantified. Identical set-ups without fungi or bacteria served as controls. All experiments were at least conducted in triplicates.

### 2.6 The bacterial distribution mediated by *Pythium ultimum* in heterogeneous soil

To investigate the bacterial distribution with hyphae in soil with different sizes, similar set-up (Figure 1C) has been used. Filled soil in the columns had a little difference; CS1, CS2, MS, and FS were combined in pairs with a 1.5 cm layer of each medium. *Pythium ultimum* was inoculated on the bottom PDA disks. The top PDA disks were removed throughout the soil after the growth of hyphae, and 500 μL of bacterial suspension was introduced from above. After 7 days of incubation at room temperature, the number of bacterial cells in soil of upper or lower layers was examined. Samples were suspended in PBS, vortexed (1 min, three times, with 30-s intervals), diluted, and spread on LB agar plates (Yang et al., 2018). Colonies were enumerated and CFU numbers were calculated. Experiments were performed in triplicates. The non-fungal or non-bacterial controls were examined similarly.

### 2.7 Biodegradation of phenanthrene in soil with different particle sizes by LW2 affected by *Pythium ultimum*

The sterilized soil of different particle sizes with a dry weight of 1 kg was accurately weighed in a sterile beaker, and a small part of it was added with 10 mL of phenanthrene stock solution (5 g L^−1^ dissolved in acetone). The soil was evenly mixed and placed in the fume hood. After the acetone was completely volatilized, it was mixed with the remaining uncontaminated soil and placed in the fume hood for more than 1 week. Due to the loss in the treatment process, the phenanthrene concentration in CS1, CS2, MS, and FS was 47.78, 46, 78, 48, 14, and 48.13 mg kg^−1^, respectively.

Except that the filled medium was replaced with 5 g contaminated soil, and the set-ups used were consistent with 2.5. *Pythium ultimum* was inoculated on the bottom agar, and a 100-μlL bacterial suspension was added from the top. Columns without inoculating microorganisms and only adding hyphae or bacteria were regarded as the control groups. The columns were cultured in dark at 25°C and destructively sampled on days 7, 14, 21, 28, and 35 to test the residual phenanthrene and bacterial count in the soil. More than three parallel samples were set at each sampling point in all experimental groups. Phenanthrene extracted was from soil according to the description by Chen and Ding (2012). The concentration of phenanthrene in samples was measured using HPLC.

### 2.8 Statistical analysis of the data

At least three parallel samples were performed for all experiments. All data were subjected to analysis of variance (ANOVA) using SPSS 25. A *p*-value<0.05 indicated that data were significant.

## 3 Results

### 3.1 Biodegradation of phenanthrene in culture medium by LW2 enhance using *Pythium ultimum*

**Figure 2:**
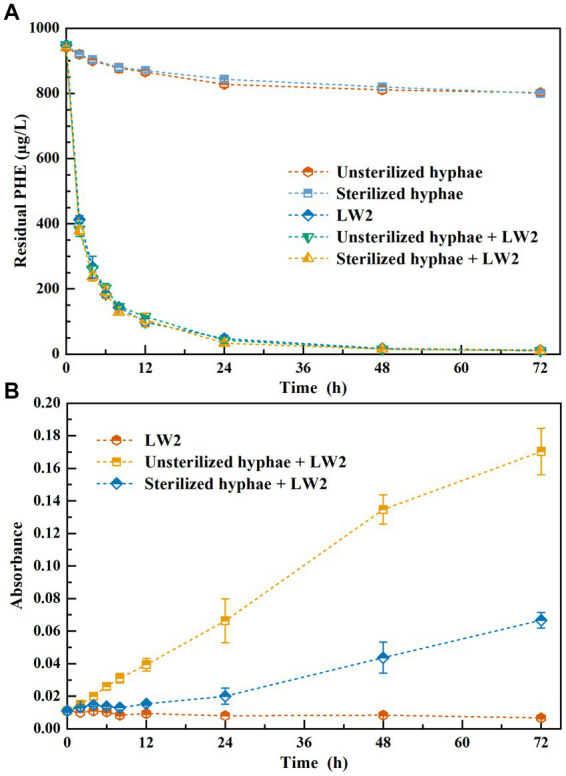
The residual phenanthrene **(A)** and the absorbance of solution at a wavelength of 600 nm **(B)** while addition of LW2sterilized, or unsterilized hyphae and both of them.

Figure 2 shows the content of residual phenanthrene and the absorbance of solution at a wavelength of 600 nm. Phenanthrene was significantly removed inoculated with LW2 (*p*<0.05), and the results of additional LW2 with sterilized or unsterilized and without hyphae showed no obvious difference (*p*>0.05). Over 87% of phenanthrene was removed within 12 h, and the residual phenanthrene was less than 13 μg L^−1^ at 72 h. The unsterilized or sterilized hyphae promoted the growth of LW2, with OD_600nm_ of 0.170 and 0.067 at 72 h, respectively. Groups without LW2, the content of phenanthrene decreased from the initial 941.41 μg L^−1^ (unsterilized hyphae) and 946.26 μg L^−1^ (sterilized hyphae) to 802.57 μg L^−1^ and 800.15 μg L^−1^. *Pythium ultimum* was unable to degrade phenanthrene. The decrease in phenanthrene was possibly due to photolysis or volatilization.

### 3.2 Migration of LW2 via fresh or old hyphae

Factors that the inoculum was fresh or old might affect the growth of hyphae. While introducing the growth of old hyphae, the diameter variation of the coverage area of mycelium is shown in Table 1. The younger inoculum had coverage radius of 19 ± 2.24 mm at 24 h, which was larger than that was formed by old hyphae. Moreover, after 3 days of incubation, both mycelia grew over the 90 mm Petri dishes. Fresh hyphae adapted to the environment and grew faster than aged hyphae.

**Table 1:** The coverage range of the hyphal network when the inoculum was fresh and old hyphae.

Time / h	Radius of hyphae /mm	
	Fresh hyphae	Old hyphae
24	19.00 ± 2.24	15.00 ± 1.41
48	35.25 ± 0.56	34.63 ± 1.71

**Figure 3:**
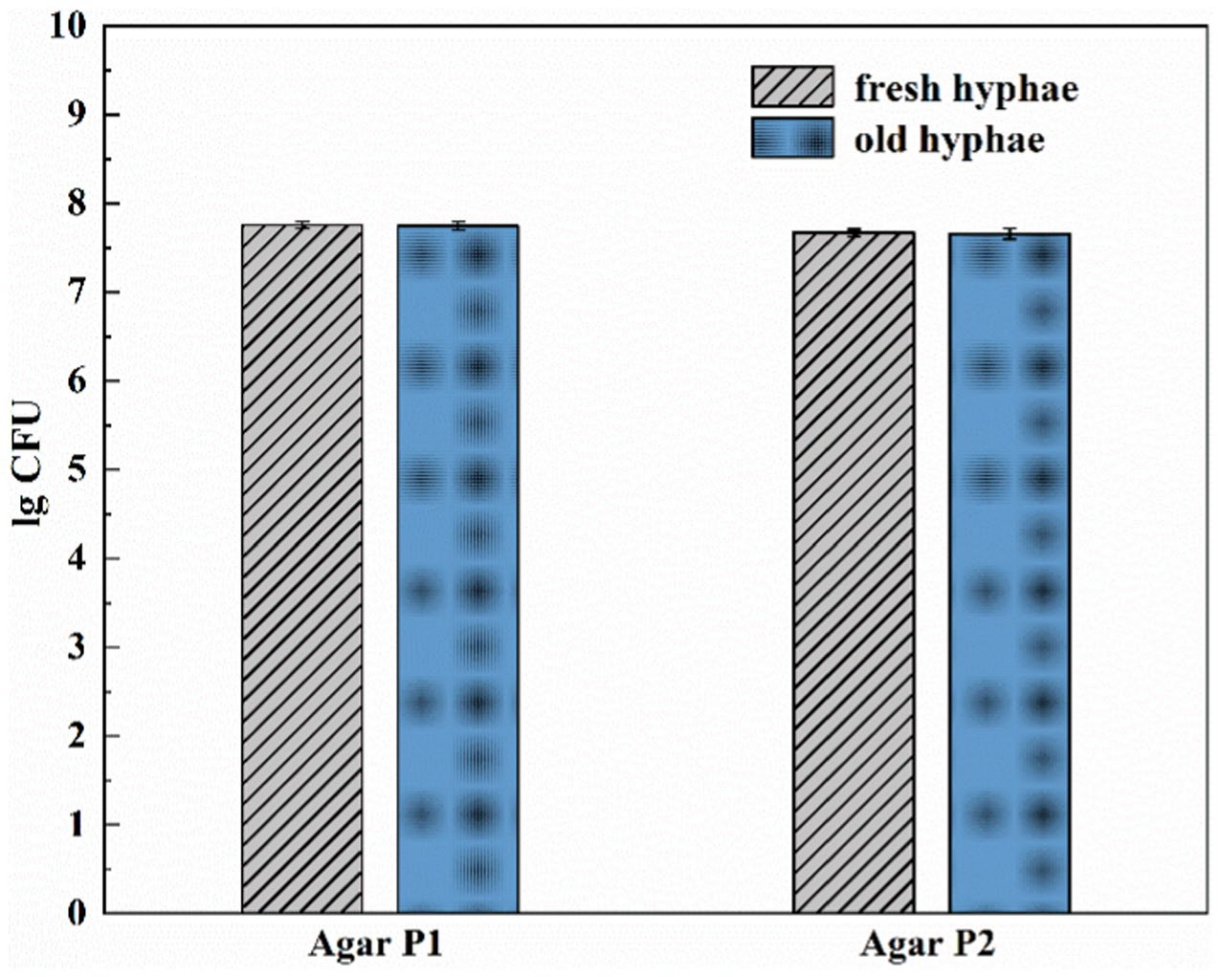
Amount of *Diaphorobacter* sp. LW2 migrated to agar P1 and P2 affected by fresh or old *Pythium ultimum* hyphae. Agar P1 and P2 were 2 mm and 12 mm away from the agar block inoculated with bacteria, respectively. Test of inoculation of LW2 when the hyphae just extended to the right PDA agar block was considered as fresh hyphae treatment. Test of inoculation of LW2 after the right agar grew full of hyphae and remained for 7 days was considered as old hyphae treatment.

It has been reported that hyphae might release nutrients as its growth may exert positive or negative effects on the bacterial movement at mycelium (Haq et al., 2018). We thus tested the migration of LW2 at newly grown hyphae and hyphae that has grown for more than a week using the set-ups, as shown in Figure 1A. Figure 3 showed the number of bacterial colonies detected on two MSM agar blocks located closer to the bacterial inoculation site (P1) and slightly further away (P2). Fresh or old hyphae showed no significant effect on the number of detected LW2 at P1 and P2 positions (P>0.05). The detected LW2 at P1 was approximately 5–6 × 10^7^ CFU ml^−1^ and 4–5 × 10^7^ CFU ml^−1^ at P2. The amount of LW2 was all in the same order of magnitude. No bacterial migration was detected in the absence of hyphae.

### 3.3 The diffusion and distribution of LW2 inoculated at the end or tip of *Pythium ultimum* hyphae

**Figure 4:**
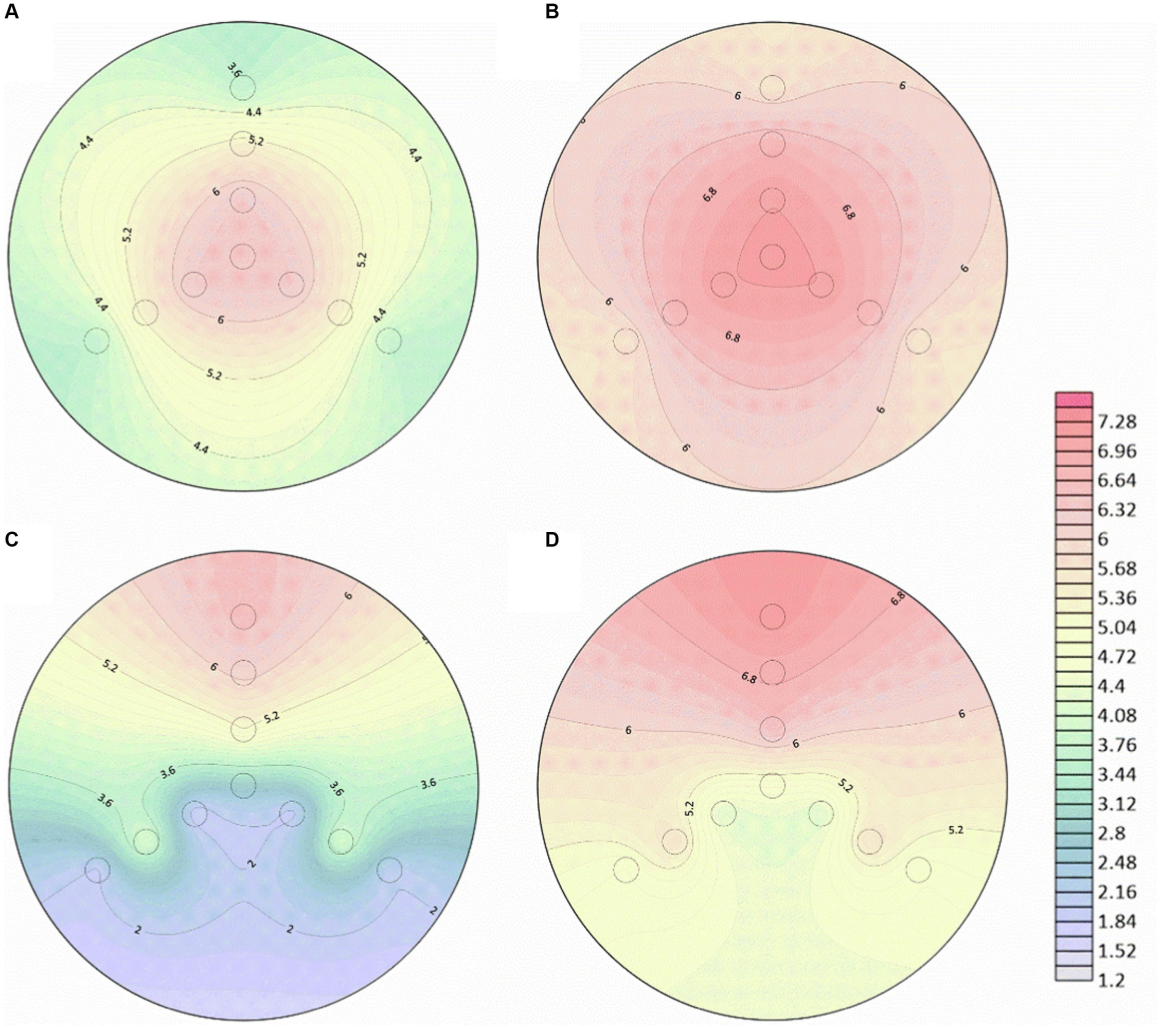
Number of bacterial cells at different positions in the mycelial network when *Diaphorobacter* sp. LW2 was inoculated at the starting position of mycelium growth **(A,B)** or hyphal tips **(C,D)**. The bacterial count test was located along the upper (U), left (L), and right (R) directions at an angle of 120 ° each. The edge (E), middle (M), and center (C) detection positions were 10 mm, 28 mm, and 46 mm away from and the hyphal inoculation point, respectively. **(A,C)** were results for 2 h; **(B,D)** were results for 48 h.

After the introduction of hyphae in the center of 150 mm PDA plates for 4 days, the mycelium covered the entire dishes. Following that, bacterial suspensions were inoculated at the end or tip of *Pythium ultimum* hyphae to investigate the diffusion and distribution of LW2 in continuous hyphae. At set times (2 h and 48 h after bacterial inoculation), samples at positions shown in Figure 1B were collected. Colonies were enumerated, and contour maps of lg CFU were drawn to analyze the distribution of LW2 in mycelium (Figure 4). When inoculating LW2 at the center position (Figures 4A,B), there was no significant difference in the detection results in the upper, lower left, and lower right directions at the same distance from the inoculation point (*p* > 0.05), and the bacterial colony numbers were all in the same order of magnitude. There were significant differences in the detection results at different distances from the bacterial inoculation site (*p* < 0.001). The detected colonies in the central position at 2 h was much greater than that in the middle and edge positions, which were 8.32–8.84 and 414–504 times higher, respectively. Over time and bacterial reproduction, CFUs in all positions increased, and the number of bacteria in the center position was approximately 2.2–2.4 and 23.73–25.36 times of that at the middle and edge positions for 48 h.

When LW2 was inoculated at the tip of the mycelium, the number of bacteria detected above was significantly greater than that in the left and right directions (*p* < 0.05), but the results in the left and right directions were not similar (*p* > 0.05). The inoculum diffused from the inoculation point to the surrounding area, but the number of bacteria detected at sites LM and RM was greater than that at positions LC and RC that was closer to the inoculation site (*p* < 0.05). The detection results at 2 h and 48 h were 108–170 and 6.8–9.1 times higher than those at the center of the left and right directions, respectively. With the absence of hyphae, no bacterial migration was detected regardless of where the bacteria were inoculated on the plate.

### 3.4 Mobilization of LW2 mediated by *Pythium ultimum* throughout soil of different particle sizes

**Figure 5:**
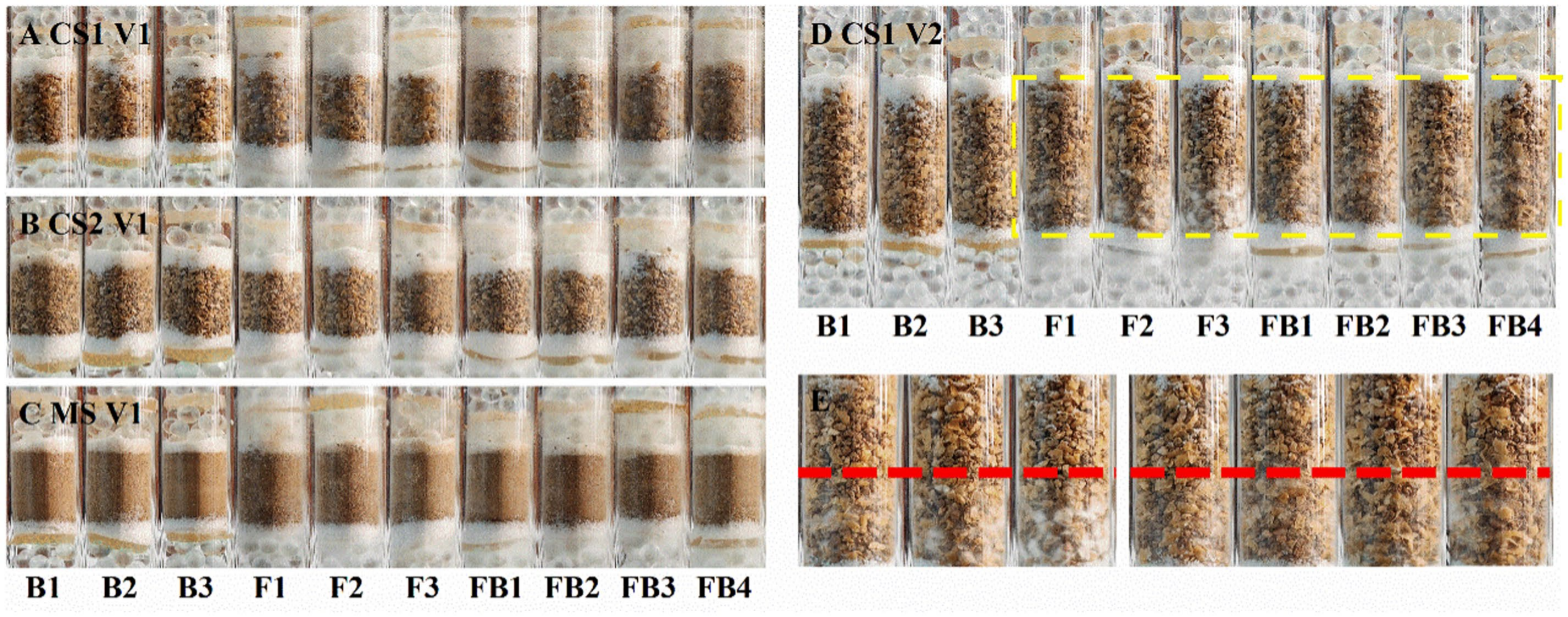
Photos of glass columns filled with soil of different particle sizes inoculated with LW2 and *Pythium ultimum* after culturation for 1 week. “FB” represented column inoculated with LW2 and *Pythium ultimum*. “F” and “B” were controls without bacteria or hyphae. **(A,C)** were photos of columns filled with CS1 (coarse sand 1, 1–2 mm), CS2 (coarse sand 2, 0.5–1 mm), and MS (medium sand, 0.25–0.5 mm) with a height of 1.5 cm. **(D)** showed CS1 columns with 3 cm soil layer. **(E)** was an enlarged image of the yellow dashed area in **(D)**. White hyphae could be clearly observed below the red dashed line in **(E)**. *Pythium ultimum* or *Diaphorobacter* sp. LW2 was inoculated onto the agar below the soil layer.

It has been proven that the strain LW2 could migrate along *Pythium ultimum* hyphae across air gaps within a centimeter range. The ability of LW2 to migrate in soil mediated by hyphae would be a favorable factor for its application in contaminated soil remediation, and we reasoned that the migration of LW2 in soil of different particle size also varied. Therefore, the travel of LW2 through coarse sand (CS1 and CS2), medium sand (MS), and fine sand (FS) in the presence of mycelium was studied. Figure 4 shows photos of columns filled with CS1, CS2, and MS cultured for 1 week. The hyphae passed through soil layers with a height of 1.5 cm within 1 week but did not pass through FS of the same height, which finally did it at the third week (Supplementary Figure S2). In the first week, *Pythium ultimum* failed to pass through the soil layers of various particle sizes with a height of 3 cm but crossed the 3 cm soil layers of CS1 and CS2 in the second week. Moreover, the hyphae in the lower soil pores could be observed in CS1 (Figures 5D,E) and MS (Supplementary Figures S1C,D) columns. Over the culturation time, *Pythium ultimum* went throughout MS and FS soil layers of a height of 3 cm at third and fourth weeks (Supplementary Figures S2A,C).

The bacteria that crossed through the filled soil via hyphae were examined. No bacteria were detected around the upper glass beads in controls. The existence of LW2 near the upper glass beads was ensured after inoculation for 2 weeks when hyphae had crossed 3 cm soil layers of CS1 and CS2 and 1.5 cm soil layers of MS. The bacteria crossing 1.5 cm thick soil layers were more than those of 3 cm and differed by approximately one order of magnitude (Table 2). In the columns filled with soil (at a height of 1.5 cm) of different particle sizes, the number of migrating bacteria detected was at a level of 10 ^6^. Moreover, in 1.5 cm FS and 3 cm MS columns, hyphae at upper PDA disks were observed, and CFUs (×10 ^4^) were 50.30 ± 4.19 and 18.30 ± 1.20. Mediated by *Pythium ultimum*, LW2 finally migrated over a distance of 3 cm in FS soil (<0.25 mm).

**Table 2:** The number of *Diaphorobacter* sp. LW2 crossing through soil with different particle sizes and thicknesses mediated by *Pythium ultimum*.

Soil thickness	CS1	CS2	MS	FS
1.5 cm	269.33 ± 18.79^a^	436.67 ± 40.78^a^	165.67 ± 11.56^a^	50.30 ± 4.19^b^
3 cm	41.37 ± 5.77^a^	56.40 ± 6.89^a^	18.30 ± 1.20^b^	7.47 ± 0.95^c^

a, b, and c were the results measured after 2, 3, or 4 weeks of cultivation, respectively (CFU, ×10 4).

### 3.5 Distribution of LW2 in soil with different particle sizes

**Figure 6:**
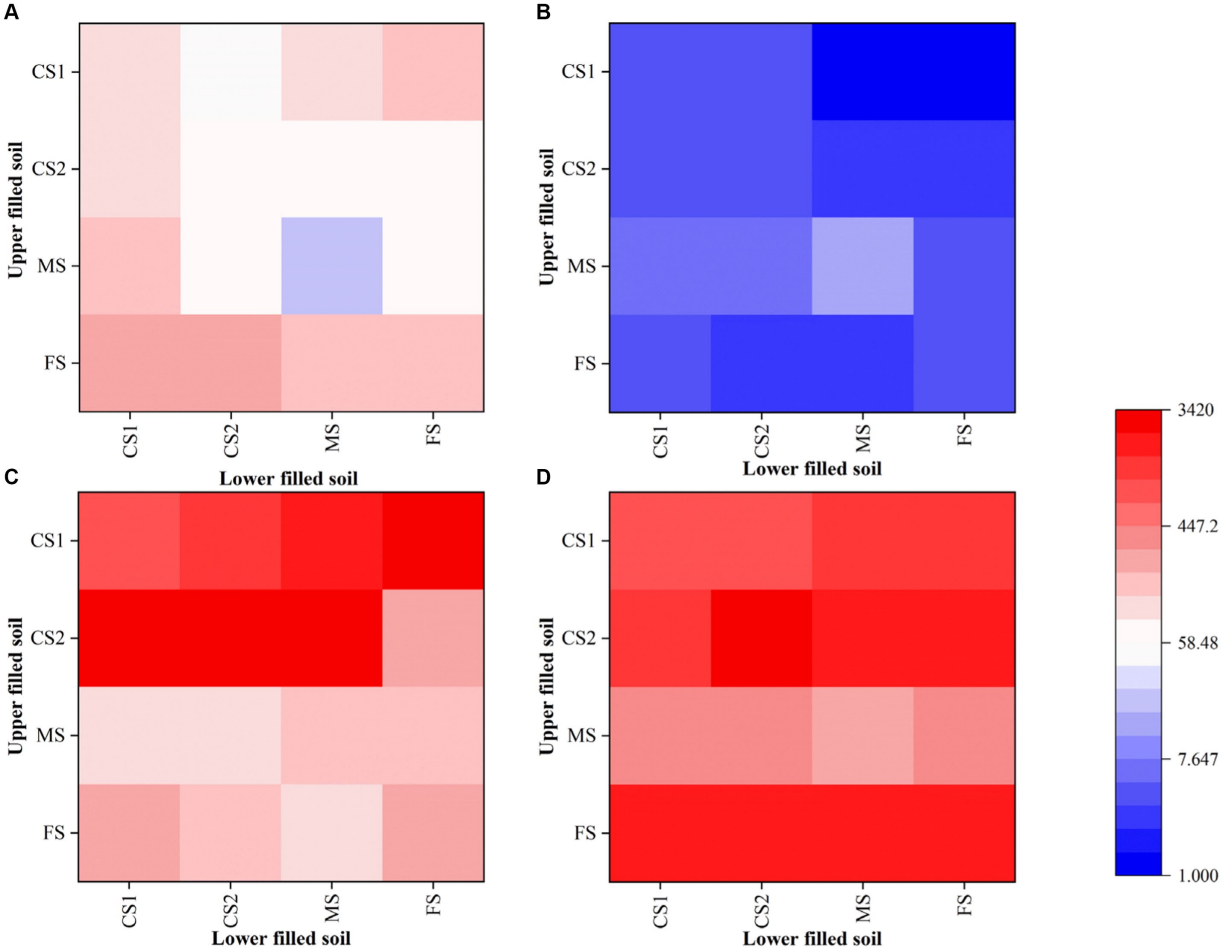
Quantity of *Diaphorobacter* sp. LW2 in soil layers with different particle sizes after bacterial inoculation (× 10 ^8^). CS1 (coarse sand 1, 1–2 mm), CS2 (coarse sand 2, 0.5–1 mm), MS (medium sand, 0.25–0.5 mm), and FS (fine sand, <0.25 mm) were pairwise combined. The x-axis and y-axis showed the types of the soil in upper and lower layers, respectively. **(A,B)** represented the results in the absence of hyphae. **(C,D)** represented the results in the presence of hyphae. **(A,C)** were number of LW2 cells in the upper soil layer, while **(B,D)** were those in the lower soil layer.

The effect of additional *Pythium ultimum* on the distribution of LW2 in homogeneous and heterogeneous soils was investigated. In soil columns of various sizes without *Pythium ultimum*, the number of bacteria detected in the lower soil layer was lower than that in the upper layer (as shown in Figures 6A,B). Soil particle size and heterogeneity significantly affected the number of LW2 in the upper- and lower-layer soil (*p* < 0.05). When the upper soil was CS1 and FS, the impact on the bacterial count in the upper soil was more significant (*p* < 0.05), and the diffusion of strain LW2 in CS1 and FS was limited. When the upper soil was MS, the influence on the bacterial count in the lower soil was more significant (*p* < 0.05). The CFU of strain LW2 in the lower soil was generally 10^7^, while in homogeneous MS, the bacterial count in the lower soil is only 1.66 × 10^8^. When the lower media were same, except for the case where the upper layer was FS, the larger the particle size difference between the upper and lower media, the more LW2 was intercepted by the upper layer.

With the presence of *Pythium ultimum*, the amount of bacteria significantly increased (*p* < 0.05), especially in the lower soil layer where the bacterial count increases by 2–3 orders of magnitude (Figures 6C,D). In columns filled with CS1 and CS2 in the upper layer, colonies from the upper layers were more than those from the lower layers. When the upper filled media was MS, the numbers of bacteria were similar in both layers, and when it was FS, 10 times bacterial cells in the lower soil layers existed compared with those in upper soils. The presence of hyphae promoted the proliferation of LW2 and altered its distribution in soil.

### 3.6 Biodegradation of phenanthrene in soil with different particle sizes by LW2 enhance by *Pythium ultimum*

The removal of phenanthrene by LW2 in soils of different particle sizes and enhancement of *Pythium ultimum* were investigated. The initial concentrations of phenanthrene in CS1, CS2, MS, and FS-contaminated soil were slightly different (*p* > 0.05), with values of 47.78, 46.78, 48.14, and 48.13 mg kg^−1^, respectively. The slight decrease (*p* > 0.05) of phenanthrene in soils without microorganisms might be related to the volatilization or photolysis. The addition of hyphae caused a decrease in phenanthrene, and the removal rates in CS1, CS2, MS, and FS were increased by 10.86, 10.57, 4.35, and 2.96%, respectively.

When LW2 was added to the soil, the residual amount of phenanthrene in CS1, CS2, MS, and FS after 21 days was 12.35, 12.74, 9.97, and 2.09 mg kg^−1^, respectively. The content of phenanthrene in soil with different particle sizes significantly decreased (*p* < 0.05). After 35 days, the remaining phenanthrene in CS1, CS2, MS, and FS were 10.93, 10.81, 7.49, and 2.10 mg kg^−1^, respectively. In liquid cultivation, LW2 almost completely degraded phenanthrene with the extension of culture time, but there was no significant change in phenanthrene for 21–35 days (*p* > 0.05). In addition, compared with 21 days, the amount of LW2 bacteria detected in soil decreased at 35 days, indicating that the physiological activity of LW2 was restricted. In the columns of *Pythium ultimum* and LW2, the residual phenanthrene in CS1, CS2, MS, and FS after 35 days was 1.35, 2.23, 2.55, and 1.41 mg kg^−1^, respectively. Compared with treatment with only LW2, the removal rates of phenanthrene were increased by 20.05, 18.36, 10.28, and 1.44%, respectively. The mycelium significantly enhanced the degradation of phenanthrene by LW2 (*p* < 0.05). Compared with columns without hyphae, the content of phenanthrene in the soil treated with hyphae and LW2 continued to decrease for 2–35 days.

## 4 Discussion

### 4.1 The effect of *Pythium ultimum* on the growth of LW2

Strain LW2 grew and propagated using phenanthrene as the sole carbon source and energy source in the absence of hyphae. As the concentration of phenanthrene decreases, bacterial reproduction was restricted. In the presence of hyphae, degradation of phenanthrene showed no significant difference (P>0.05), but the biomass increased. Hyphae would secrete some compounds during its growth, and some of them could be utilized for bacterial growth (Boersma et al., 2010; Haq et al., 2018). Gandhi and Weete (1991) have been reported the production of polyunsaturated fatty acids such as arachidonic acid and eicosapentaenoic acid by *Pythium ultimum*, which could serve as nutrients for bacterial growth. The promoting effect of sterilized hyphae is slightly weaker, which is likely due to the high temperature accelerating the release of compounds secreted by hyphae, resulting in a decrease in the amount of nutrients added to the culture medium. *Pythium ultimum* hyphae could enrich and adsorb phenanthrene from solutions (Supplementary Figure S3). Furuno et al. (2012) found that phenanthrene could accumulate in *Pythium ultimum* lipid vesicles and be transported in hyphae. The adsorption of phenanthrene by *Pythium ultimum* hyphae and the promotion of bacterial growth are beneficial to the bioremediation of actual phenanthrene-contaminated soil.

### 4.2 Transport of LW2 by *Pythium ultimum* on PDA plate

We have demonstrated in previous studies that the strain LW2 could migrate within a centimeter range in Petri dishes through the water channels provided by *Pythium ultimum* (Li et al., 2022). In this study, we compared the migration of LW2 in fresh and old hyphae and found that the difference was very small. Warmink and Elsas (2009) reported that the migration of inoculant strains, *Dyella japonica* BS021 and *Burkholderia terrae* BS001, only occurred at the growing hyphal front of *Lyophyllum* sp. strain Karsten, and no bacterial translocation was observed along hyphae grown over a week at any directions. However, *Achromobacter* sp. SK1 was found to move at old hyphae of *Fusarium oxysporum* Fo47 during their interaction (Kohlmeier et al., 2005). Moreover, Yang et al. (2018) found that the strain BS001 was able to migrate along the old hyphae of *Lyophyllum* sp. strain Karsten while investigating its movement at various acid pH levels. The tropic movement of BS001 was due to its chemotactic response toward oxalic acid that *Lyophyllum* sp. strain Karsten exudated (Haq et al., 2018). In our experiment, the new and old hyphae showed similar impact on the migration of LW2. Meanwhile, no matter inoculum was introduced at the front or end of the hyphae, the bacterial distribution in mycelial networks gradually became uniform over time. This was likely due to *Pythium ultimum* secreting different compounds, and LW2 did not receive special signal resulting its isotropic movement. The diffusion of bacterial LW2 along the mycelium in this article was largely related to the diffusion time and bacterial concentration, which was consistent with the research results by Wick et al. (2007).

The detection of bacteria in the opposite direction of mycelial growth proved that LW2 that translocated via the liquid channels provided by hyphae along or against the direction of mycelium growth. The number of bacteria detected at position LM and RM for LW2 was greater than that at position LC and RC closer to the hyphal inoculation location (Figures 4C,D). Overall, hyphae grew radially from the inoculation site to the surrounding area on PDA plates. The intersection of hyphae was relatively less near the inoculation site but increased with hyphal growth, and the water channels formed by the liquid film around the hyphae were interconnected (Heaton et al., 2012). Therefore, fewer bacteria migrated to position LC and RC but increased with time.

Currently, several different mechanisms have been reported by which hyphae enhance the migration of moving bacteria. For hydrophobic hyphae, bacteria anchor on the surface of the hyphae through capillary forces, van der Waals forces, and cross-linking forces and passively migrate as the hyphae grow and elongate (Gu et al., 2017). Bacteria may also attach to the surface of hyphae using the organic matter secreted by the hyphae as a carbon source and energy source for growth and reproduction and form biofilms on hyphae and passively migrate with fungal growth. With this mechanism, bacteria could migrate only when inoculated at the tip of hyphae, and migration occurred along the direction of hyphal growth (Frey-Klett et al., 2007; Warmink and Elsas, 2009; Nazir et al., 2010a,b). For hydrophilic hyphae, a liquid film was wrapped around it, and moving bacteria could actively migrate along the water channel based on their own mobility (Kohlmeier et al., 2005; Wick et al., 2007). *Diaphorobacter* sp. LW2 can be detected along the growth direction or in the opposite direction of *Pythium ultimum* hyphae, indicating that LW2 can migrate in accordance with the “inherent mobility hypothesis.”

### 4.3 Mobilization of LW2 by *Pythium ultimum* mycelium in soil of different particle sizes

The moisture content of experimental soil was appproximately 10%, and the saturation was in range of 15.7 to 20.8%. The discontinuous distribution of water in the soil limits the active migration of bacteria. The diffusion of bacteria in soil usually only occurred when the soil moisture was abundant and the water potential was > −0.05 MPa (Gülez et al., 2010). The water potentials for CS1, CS2, MS, and FS were −2.38, −2.45, −3.07, and −3.07 MPa, which did not meet this limitation. Therefore, LW2 has not been detected at the top in the absence of hyphae.

When crossing soil layers of different particle sizes in hyphae, the amount of mobilized bacteria reduced with the decrease of particle size, with the exception of CS1. Kravchenko et al. found that, when the initial water contents were relatively low, *E. coli* introduced into 4–6 mm soil aggregates and preferred to distribute in pores of 15 μm than pores >100 μm (Kravchenko et al., 2013). The introduced microorganisms tended to inhabit in the liquid habitat maintained by capillary force at the gaps and particle contacts of the medium. LW2 existed not only in the liquid membrane of the hyphae but also in the liquid habitat at the pores of the soil after migrating with the *Pythium ultimum*. It has been reported that microorganisms often colonize at the interface or interior of the aggregates in soil (Xia et al., 2022). Moreover, the bacteria would migrate to other locations via mycelium networks after proliferation. CS1 columns provided less liquid habitat compared with soils with smaller particle size, leading to fewer opportunities for microbial colonization. As a result, the number of bacteria migrating to the top in the CS1 column was less than in CS2. In the columns packed with soils of sizes <1 mm, the positive correlation between the number of migrating bacteria and the particle size was possibly due to mycelial growth. In soils with smaller particle sizes, hyphae need to bypass further paths to reach the same distance, which takes longer.

LW2 succeeded translocation in soil by the mobilization of *Pythium ultimum* and took longer time in soil of smaller particle. The migration of bacteria in unsaturated soil at a macroscopic scale (>10 mm) generally only occurred when the soil was close to saturation (Or et al., 2007), while *Pythium ultimum* hyphae bridged soil pores filled with air, and the liquid film around hydrophilic hyphae provided a continuous water channel for strain LW2, allowing the bacteria to migrate at a macroscopic scale in coarse, medium, and fine sandy soils. As reported by Yang et al. (2018), *paraburkholderia terrae* BS001 also achieved migration in soil under the mediation of mycelium, and the promotion effect of mycelium on bacterial migration was more significant in unsaturated soil with higher water content. Microorganisms tend to form colonies or biofilms at the corners of soil pores, gathering to form microbial hotspots (Dechesne et al., 2008; Kuzyakov and Blagodatskaya, 2015). Although the number of microorganisms in the soil could reach an average of 10^7^–10^12^ cells per gram of soil, these microbial environments only account for less than 1% of the total soil volume (Watt et al., 2006). The effective contact between degrading bacteria and toxic compounds was a prerequisite for the bioremediation of polluted soil. The *Pythium ultimum* served as a bridge for the mobile strain LW2, allowing it to diffuse in unsaturated soil, which could increase the frequency of microbial contact with substrates, improve the bio-accessibility of pollutants in soil, and provide feasible methods for improving the remediation effect of polluted soil.

### 4.4 Redistribution of LW2 in heterogeneous soil by *Pythium ultimum*

The remaining water in unsaturated soils generally formed a liquid habitat in the corners and crevices among particles or thin water films on rough surfaces (Or et al., 2007). The water environment was discontinuous and fragmented, and the intermittent liquid films were not available for free bacterial transporting, resulting in the interception of strain LW2. Bacteria passively transported under the action of water flow in unsaturated environments (Yang and van Elsas, 2018). In the absence of mycelium, the bacterial suspension migrated downward under the action of gravity after entering the soil. The order of soil water potential was CS1 > CS2 > MS > FS, indicating that the water flow in the soil with large particle size was subjected to less resistance, so it was easier to migrate downward. However, the saturation in coarse sandy soil was lower, and microorganisms were easily adsorbed at the air-water interface (Wan et al., 1994; Schafer et al., 1998). Therefore, under multiple effects, the number of bacteria intercepted by coarse and fine sandy soil in homogeneous soil is greater than that in MS. In addition, droplets in pores are also affected by capillary forces. Guber et al. (2009) reported that water flow driven by capillary forces was the main mechanism of *E. coli* entering air-dry aggregates. Supplementary Figures S4, S5 show the longitudinal and transverse sections of soils with different particle sizes, respectively, showing that the pores in soils with larger particle sizes were larger. Kravchenko et al. (2013) found that water had limited entrance into large biological pores when it reached them from smaller pores and mainly influenced by capillary forces. The size of soil pores changes at the interface of heterogeneous soil. According to the relationship between capillary force and pore radius, water droplets in coarse sandy soil experience less capillary force than in MS and FS. Therefore, when the pores in the lower soil are larger than those in the upper soil, it is difficult for bacteria that introduced in MS and FS to enter the larger sized lower soil and remain in the upper soil.

In the presence of *Pythium ultimum*, the proportion of bacteria in the upper soil decreased from 58.78–99.10% to 0.05–69.50%, promoting the migration of mobile bacteria LW2 in heterogeneous soil. Hydrophilic hyphae penetrated the soil layer, bridging air filled soil pores. The liquid film surrounding the hyphae provided a continuous water channel for the migration of LW2, changing its migration mode from passive transportation to autonomous migration. The energy state of water in soils with smaller particle sizes is lower, and bacterial movement was more restricted. Therefore, when the particle size of the upper layer medium was smaller, the promotion effect of *Pythium ultimum* on bacterial migration was more significant.

Based on the above results, we speculated that in the presence of hyphae, the introduced bacteria migrated from the inoculation position to all directions, possibly all the way down to the lower soil layer along with *Pythium ultimum* hyphae or colonized in suitable habitats in the upper soil layer. The proliferated bacteria then spread to other locations with the hyphae, ultimately making the bacteria to be distributed throughout the soil. The particle size of the soil in the natural environment was often uneven, which caused difficult migration of added bacteria on the basis of greatly varied distribution. The spatial network formed by mycelium could connect different soil pores of different sizes, providing an effective migration pathway for degrading bacteria and improving the uniform distribution of bacteria in heterogeneous soil, offering available assistance for the remediation of contaminated soil.

### 4.5 The removal of phenanthrene by LW2 in soils and enhancement of *Pythium ultimum*

**Figure 7:**
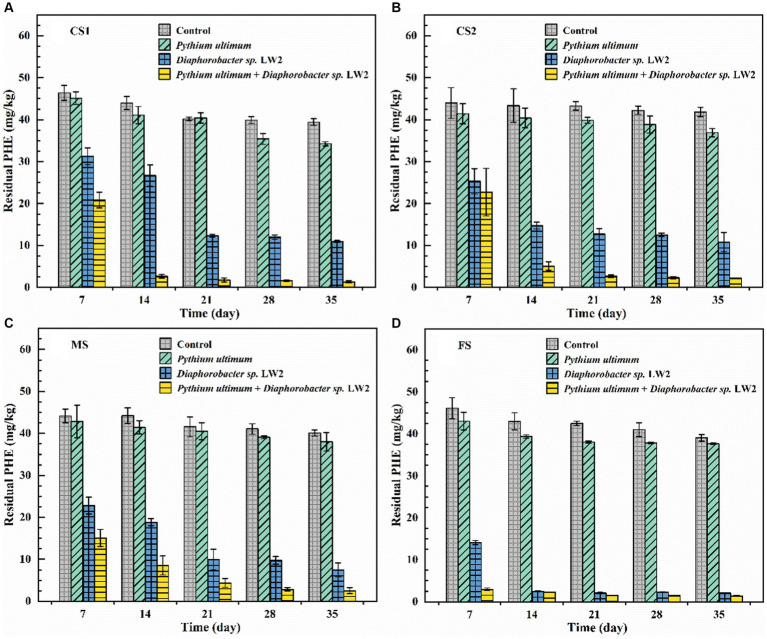
The content of phenanthrene in coarse sand (**A**: CS1, **B**: CS2), medium sand (**C**, MS), and fine sand (**D**, FS) with no treatment, only adding *Pythium ultimum* or *Diaphoractor* sp. LW2 or adding both *Pythium ultimum* and *Diaphoractor* sp. LW2.

It was observed that *Pythium ultimum* hyphae penetrated the soil layer in the column and continued to grow outside the soil (Figure 5). *Pythium ultimum* could adsorb and actively transport phenanthrene through mycelium (Furuno et al., 2012). The decrease of phenanthrene in soil can be attributed to the adsorption and transportation of phenanthrene by mycelium. Meanwhile, the concentration of phenanthrene varied more in coarse sand soil, which corresponded to the result that it took shorter time for mycelium to cross the coarse sand soil. LW2 was added to the soil and phenanthrene was degraded. After 21 days, the amount of phenanthrene decreased by more than 70%. However, due to the limited migration of bacteria in unsaturated soil, LW2 cannot sufficiently contact to pollutants in the soil, making it difficult to effectively degrade the pollutants. Phenanthrene was not further degraded for 21–35 days. Compared with the influence of soil environmental factors on the biological activity of degrading bacteria, the poor accessibility of polycyclic aromatic hydrocarbons further limits the microbial bioremediation of polluted soil (Puglisi et al., 2007). Improving accessibility between pollutants and microorganisms is crucial for improving bioremediation efficiency (see Figure 7).

Similar to the extension of plant roots, which can promote the distribution of microorganisms in soil, the mycelial network in pores increases the contact between degrading bacteria and pollutants (Aprill and Sims, 1990). In the soil where hyphae and LW2 coexisted, the removal rate of phenanthrene in soil of different particle sizes was above 94% after 35 days. *Pythium ultimum* promoted the diffusion of LW2 in soil by connecting soil pores and providing effective pathways for bacterial movement, thereby improving the removal of phenanthrene in soil. Meanwhile, due to the fact that *Pythium ultimum* could adsorb phenanthrene and actively transport it, the bioavailability of phenanthrene has also increased (Furuno et al., 2012; Schamfuß et al., 2013). As the cultivation time prolonged, water in the soil evaporated, the suitable microbial environment decreased, and the number of bacteria in the soil gradually decreased. When mycelium was present, the number of LW2 colonies in soil with different particle sizes was always more than that in soils without hyphae (Supplementary Figure S6). The presence of hyphae delayed the decay of bacterial cells in soil and prolonged the biological function of soil, which was consistent with the research results by Nazir et al. (2010a,b). In dry soil, hyphae could transport water from sufficient areas to impoverished areas based on their own growth patterns, which promotes bacterial migration (Heaton et al., 2012). Moreover, the redistribution of soil moisture by hyphae can enhance soil biological activity and metabolic function (Guhr et al., 2015). In soil at various acid pH levels, mycelium *Lyophyllum* sp. strain Karsten promoted bacterial migration and survival, although lower pH levels showed negative effects (Yang et al., 2018).

Bacteria and fungi coexisted in the soil, either not interfering with each other, competing with each other, or cooperating with each other, to jointly maintain the ecological function of the soil (Nazir et al., 2010a,b). The soil matrix was a natural barrier for bacterial migration, and most bacteria did not possess the mycelial growth pattern of filamentous organisms such as actinomycetes and were intercepted in the soil (Schafer et al., 1998). Mycelial organisms, especially fungi, could penetrate air-filled soil pores and transport carbon containing compounds over long distances, providing nutrients for cell growth (Heaton et al., 2012). The participation of mycelial microorganism *Pythium ultimum* enhanced the diffusion and survival of LW2 in soil, which was reflected in soils of different particle sizes. The combination of *Pythium ultimum* and *Diaphoractor* sp. LW2 could improve the bioremediation efficiency of phenanthrene-contaminated soil, which had great potential for application in the remediation of actual contaminated soil.

### 4.6 The prospect of future research

In this study, we discussed the enhanced migration and biodegradation of LW2 in soil by *Pythium ultimum* in a sterile constant temperature and humidity environment. The actual environment would be more complex. Changes in organic matter, temperature, and moisture in the soil may have unknown effects on the growth, migration, and degradation behavior of mycelium and LW2. More comprehensive research was needed for the practical remediation application of polluted soil. A large number of microorganisms in the soil maintained the ecological function of the soil through mutual interaction. Introducing new microorganisms into the soil might cause changes in the existing biological community. Studying the interactions and community changes among microorganisms could provide a better understanding of the soil environment and had profound implications for the remediation of contaminated soil.

## 5 Conclusion

In conclusion, this study confirmed that the movement of *Diaphorobacter* sp. LW2 along with *Pythium ultimum* hyphae was in and against hyphal growth directions, and bacterial cells were gradually distributed evenly in mycelial networks over time. By hyphal transportation, strain LW2 could move in unsaturated soil with different particle sizes in the range of centimeters. The mycelium throughout soil pores reduced the limitation for LW2 of entering larger pore soils from fine soil. In addition, the mobilization of hyphae in uneven soil regulated the distribution of introduced bacteria. The enhancement of mycelium on the growth and migration of LW2 promotes the removal of phenanthrene by LW2 in soils of different particle sizes. Hyphal transportation with pollutant-degrading bacteria may play an important role in the bioremediation of contaminated soil.

## Data availability statement

The raw data supporting the conclusions of this article will be made available by the authors without undue reservation.

## Author contributions

JLi: Investigation, Writing – original draft. MH: Conceptualization, Supervision, Writing – review & editing. JLv: Validation, Writing – review & editing. RT: Software, Writing – review & editing. RW: Software, Writing – review & editing. YY: Data curation, Writing – review & editing. NL: Conceptualization, Project administration, Supervision, Writing – review & editing.
