# Supplementary material for: Enhancement on migration and biodegradation of Diaphorobacter sp. LW2 mediated by Pythium ultimum in soil with different particle sizes
Source: Front Microbiol. 2024 May 22;15:1391553. doi: 10.3389/fmicb.2024.1391553 (PMC11150788; doi:10.3389/fmicb.2024.1391553)
Supplement: Supplementary file 1 [file Table_1.docx]

# Supplementary Information

**Mobilization of *Diaphorobacter sp*. LW2 mediated by *Pythium ultimum* in soils with different particle size.**

**Jialu Li ^1, 3^, Mei Hong ^1, 3^, Jing Lv ^1, 3^, Rui Tang ^1, 3^, Ruofan Wang ^1, 3^, Yadong Yang ^4^, Na Liu** **^2^***

^1^ *Key Laboratory of Groundwater Resources and Environment, Ministry of Education, College of New Energy and Environment, Jilin University, Changchun 130021, China*

^2^ *Department of Ecology, College of Life Science and Technology, Jinan University, Guangzhou, 510632, Guangdong, China*

^3^ *National and Local Joint Engineering Laboratory for Petrochemical Contaminated Site Control and Remediation Technology, Jilin University, Changchun 130021, China*

^4^ *School of Environmental Science and Engineering, Jiangsu Engineering Research Center of Biomass Waste Pyrolytic Carbonization & Application, Yancheng lnstitute of Technology, Yancheng 224051, China*

Corresponding author:

Na Liu

E-mail Address: [liuna@jnu.edu.cn](mailto:liuna@jnu.edu.cn)

Table S1 Information on soil sample moisture content, organic carbon content, and porosity.

|  | Water content ^a^ (wt %) | C_org_ ^a^ (wt %) | Porosity ^b^ |
| --- | --- | --- | --- |
| CS1 | 3.18 ± 0.07 | 0.23 ± 0.02 | 0.50 ± 0.01 |
| CS2 | 3.33 ± 0.02 | 0.33 ± 0.01 | 0.49 ± 0.00 |
| MS | 3.45 ± 0.02 | 0.35 ± 0.02 | 0.49 ± 0.02 |
| FS | 3.50 ± 0.03 | 0.45 ± 0.02 | 0.48 ± 0.01 |

^a^ Measured indexes of initial soil.

^b^ In mobilization experiments, soil samples were mixed with 5% (w/w) wheat bran, and the porosities were shown above. The water content of the experimental soil was adjusted to ~10 wt %.





**Figure S1** Photos of glass columns consist of 3cm soil layer of CS1, CS2 and MS cultured for two weeks. "FB" represented column inoculated with LW2 and *Pythium ultimum*. "F" and "B" were controls without bacteria or hyphae. (A), (B) and (C) were photos of CS1, CS2 and MS with a height of 3 cm. (D) was an enlarged image of the yellow dashed area in (C). White hyphae could be clearly observed below the red dashed line in (D).





**Figure S2** Photos of *Pythium ultimum* hyphae throughout medium and fine soil. a and b were obtained at third week, and c was captured after four weeks inoculation. The thickness of MS(A) and FS(C) layers was 3cm, while in (B) the soil thickness of FS was 1.5cm. "FB" represented column inoculated with LW2 and *Pythium ultimum*. "F" and "B" were controls without bacteria or hyphae.





Figure S3 The adsorption of phenanthrene by unsterilized or sterilized hyphae.





Figure S4 Longitudinal section diagram of CS1 (Coarse sand 1, 1-2mm), CS2 (Coarse sand 2, 0.5-1 mm), MS (Medium sand, 0.25-0.5 mm) and FS (Fine sand, ＜0.25mm).





Figure S5 Transverse section diagram of CS1 (Coarse sand 1, 1-2mm), CS2 (Coarse sand 2, 0.5-1 mm), MS (Medium sand, 0.25-0.5 mm) and FS (Fine sand, ＜0.25mm).





Figure S6 The bacterial count of *Diaphoractor sp*. LW2 in coarse sand (A: CS1, B: CS2), medium sand (C, MS), and fine sand (D, FS) with or without *Pythium ultimum.*
